# Heterogeneous Catalysis to Drive the Waste-to-Pharma Concept: From Furanics to Active Pharmaceutical Ingredients

**DOI:** 10.3390/molecules26216738

**Published:** 2021-11-08

**Authors:** Rafael Luque, Zeid A. ALOthman, Alina M. Balu, Leonid Voskressensky

**Affiliations:** 1 Departamento de Química Orgánica, Campus de Rabanales, Universidad de Córdoba, Edificio Marie Curie (C-3), Ctra Nnal IV-A, Km 396, 14014 Cordoba, Spain; qo2balua@uco.es; 2 Chemistry Department, College of Science, King Saud University, P.O. Box 2455, Riyadh 11451, Saudi Arabia; zaothman@ksu.edu.sa; 3 Department of Chemistry, Peoples Friendship University of Russia (RUDN University), 6 Miklukho Maklaya St., 117198 Moscow, Russia; lvoskressensky@yandex.ru

**Keywords:** heterogeneous catalysis, waste-to-pharma, furanics, APIs

## Abstract

A perspective on the use of heterogeneous catalysis to drive the waste-to-pharma concept is provided in this contribution based on the conversion of furanics to active pharmaceutical ingredients (APIs). The provided overview of the concept in this perspective article has been exemplified for two key molecule examples: Ancarolol and Furosemide.

Society faces a daunting future in terms of water, food and resource scarcity. This has been evidenced by recent studies showing a significant diminishing of fossil fuel resources, the increasing generation of waste, as well as the expected increase in population in future years. Facing these challenges is not an easy task. A multidisciplinary team effort from many disciplines is needed to develop suitable alternatives for a more sustainable society able to deal with these important issues.

Waste is currently one of these alarming problems the planet is experiencing and will face in future years. In Europe, waste generation was reported to be over 2.25 billion tons in Western European Countries between 1998 and 2001 as well as 550 million in Eastern Europe Candidate Countries [1]. Main sources of such waste were construction (31%), mining and quarrying (15%) and most importantly, agricultural and forestry waste, which accounted for roughly 30% of the total generated waste. Agricultural and forestry waste residues currently find somewhat limited uses and exploitation different than burning, field rotting and/or composting, being under-considered despite their huge potential to be valorised.

Lignocellulosic residues (typically from tree debranching and cutting, left-overs from crops, municipal residues from packaging, etc.) comprise three markedly different fractions: hemicelluloses (30%), cellulose (45%), and lignin (25%) [2], which can potentially be isolated separately (more difficult for hemicelluloses and cellulose) and processed to valuable products.

Derived from C5–C6 fractions, furanic compounds have recently attracted significant techno-economic considerations due to their production capabilities from non-edible parts of lignocellulosic biomass to produce fuels, chemicals and materials [3]. For this reason, some have been listed by the U.S Department of Energy as one of the top 12 and top 30 potential chemical building blocks [4]. In particular, Avantium’s YXY^®^ process produces furandicarboxylic acid (FDCA) for the development of the new generation of bio-plastics PEF (Scheme 1) [5]. FDCA production process primarily involves the dehydration of carbohydrates (C6 and C5 sugars) into alkoxymethyl furfurals (RMFs), being an intermediate mixture essentially containing methoxymethyl furfural (MMF), furfural (FA) and 5-hydroxymethyl furfural (HMF) in its composition. Besides leading to various furanics and other platform chemicals, the Acid Catalysed Dehydration (ACD) process essentially leads to the production of an unavoidable side stream residue called humins (Scheme 1).

Humins are polyfuranic macromolecule mixtures with minor quantities of furanic derivatives retained in their structure [6]. The chemical structure of humins is highly complex and largely depends on the type of feedstock, operating conditions and the functional groups associated with them (Scheme 2) [7,8]. Despite its existence for many decades, humins have been mostly employed as residues in low-value applications such as combustion and gasification [9]. With the primary aim of valorising biorefinery side streams to improve the bio-based economy, innovative potential applications for humins as renewable raw materials have been identified mainly in catalysis, water purification, matrix of impregnation materials, CO_2_ sequestration and energy storage [6,10,11], with very few reports to date on the valorisation of humins towards valuable chemicals production [12,13]. A recent report by Hallet et al. discloses the use of ionic liquids (ILs) for the production of humins in view of applications as valuable carbonaceous materials for antimony removal [14]. Additionally, we recently reported a plausible structure for humins obtained via several hydro/oxy-deconstruction strategies [15], following previous reports on structural characterisation of humins [8,14,16,17,18].

New and alternative ways to synthesize APIs are largely needed in the EU due to their generally complex synthetic processes (typically 6–10 synthetic steps) and multiple rounds of quenching, separation and purification [19]. Neither innovator drug companies nor generic manufacturers have economic incentives to develop such novel, cost-saving alternative routes. Importantly, continuous flow technologies, combined with nano(bio)catalysis are highly advantageous for these purposes. Such benefits include better control of reaction conditions, which is especially advantageous in the case of highly reactive compounds such as those derived from biomass, the possibility of scaling up (of high interest and novelty in the waste-to-pharma concept) as well as less issues in catalyst separation (it stays after every run in the fixed bed reactor) and additional intermediate separation and purification steps. Lastly, continuous flow processes allow gas and/or product/byproduct removal during the reaction to not interfere in the proposed chemistries.

The identification of manufacturing routes that utilize the lowest-cost raw materials (e.g., humins) and most efficient tools available (e.g., continuous flow processes, photoredox catalysis) to make this a future reality, starting with the synthesis of two relevant APIs fully from humins (Ancarolol and Furosemide) will be reported in due course. A revolutionary and innovative approach for the valorisation of furanic-containing humins to valuable biofuranics with biological activities has been recently proposed by our group, driven by heterogeneous catalysis. All reactions are performed using low environmental impact technologies, including mechanochemistry and nano-(bio)catalysis as well as continuous flow processes in view of a future potential scaling-up (Figure 1, overall concept).

The first proposed relevant API synthesis deals with the preparation of atenolol analogues (antihypertensive drugs) from furanics derived from humins. Atenolol is an extensively prescribed API beta-blocker to treat high blood pressure [20] with a global market value of billion euros [21], being in the top 1% of drugs prescribed for patients worldwide (over 30 million only in the USA in 2015 and expected to grow by 4.94% by 2023 [22]). Importantly, since 2017, Atenolol has been listed on the FDA Drug Shortages database, making it imperative to search for chemical analogues that can provide the required biological activity without major secondary effects.

Ancarolol derivatives (Scheme 3) have been selected as target molecules, being analogous beta-adrenergic blocking agents, due to their relevance and remarkably interesting compatible structure and biological activity featuring a furanic ring coupled to an *ortho*-aminophenol derivative linked to a potential glycerol-derived tail [23]. Another potential synton (3-(*tert*-butylamino)-1,2-propanediol) that could be employed in the synthesis of the drug is a chemical intermediate utilized in the industrial chiral synthesis of related *β*-blockers—i.e., (*S*)-timolol—employed in the treatment of various cardiovascular disorders such as hypertension, angina pectoris and cardiac arrhythmia [24].

The proposed synthetic process involves four steps in principle, including the activation of the primary OH group for the final reaction, as illustrated in Scheme 3. The first step (**1**) involves a simple and previously reported one-pot synthesis of aryloxypropanediols using glycerol as starting material with various phenols including 2-methoxyphenol (99% conversion, 8 h, 110 °C) [25] however unreported for 2-aminophenol. Interestingly, such reaction generated an additional unexpected benzoxazine API-type product from the cyclisation of 2-aminophenol and glycerol [(3,4-dihydro-2*H*-benzo[*b*][1,4]-oxazin-3-yl)methanol, Scheme 4) with promising biological activities [26]. The intramolecular cyclisation reaction may involve OH activation taking place under mechanochemical conditions and the presence of potassium carbonate.

Benzoxazines are core motifs in relevant APIs including apararenone [27], elbasvir [28], and etifoxine [29].

Preliminary results indicate that furfural in the second step (**2**) can also be successfully coupled with aromatic amines (aniline, benzylamine as well as the obtained 3-(2-aminophenoxy)propane-1,2-diol in step **1**) under pulsed laser/LED irradiation using plasmonic systems (e.g., Au and Ag-based TiO_2_ catalysts) [30] via most plausible oxidative amidation (oxidation of the aldehyde group in furfural to carboxylic acid and addition of amine to form the amide) as previously reported by our group [31]. Subsequent steps can lead to a final ancarolol derivative in which more than half of the molecule is derived from renewable feedstocks (glycerol and humins). Preliminary calculations pointed to an atom economy of the whole synthetic process over 70%, with an E-factor of 7 [30], far from classical values (25–100) from the pharmaceuticals industry.

The other example for API synthesis is Furosemide. Furosemide is an extensively prescribed API with interesting biological activities in the treatment of fluid build-up (loop diuretic) due to heart failure, liver scarring, or kidney diseases [32], being in the World Health Organisation’s List of Essential Medicines as most effective and safest required in a health system [33]. The structure of Furosemide (Scheme 5) can be synthesized in three steps in a relatively simple way (also under continuous flow conditions), starting from furfural/furanics.

The proposed synthesis involves the reductive amination of furanics (including furfural and HMF) using a simple and mild reductive amination catalysed by Rh/TiO_2_ materials (94% yield from furfural to furfurylamine using 0.5%Rh/TiO_2_, 2 h reaction, 100 °C), similar to those reported to be most effective in such reaction [34]. Results are currently under translation into the continuous flow using H-Cube reactors [35,36]. Additionally, 2,4-dichlorobenzoic acid has been reacted in two steps (first SO_3_HCl, then ammonia) to form the chloro-substituted sulfamoylbenzoic acid that will eventually react with furfuryl amine to yield Furosemide (for furfural) and derivatives.

We hope the present contribution can stimulate scientists to further develop the waste-to-pharma concept and look forward to witnessing further developments on the topic in the years to come, all for a more sustainable future for the betterment of humankind.

## Figures and Tables

**Scheme 1:**
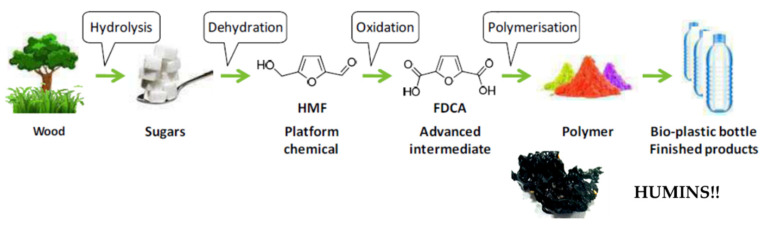
Avantium’s YXY^®^ process for PEF production generates two major side products: methyl levulinate and humins.

**Scheme 2:**
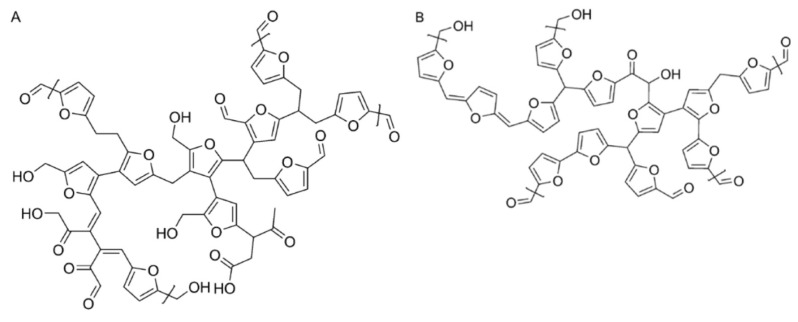
Models representing humin fragments from (**A**) glucose-derived humins; (**B**) fructose-derived humins. Reproduced from ref. [8].

**Figure 1:**
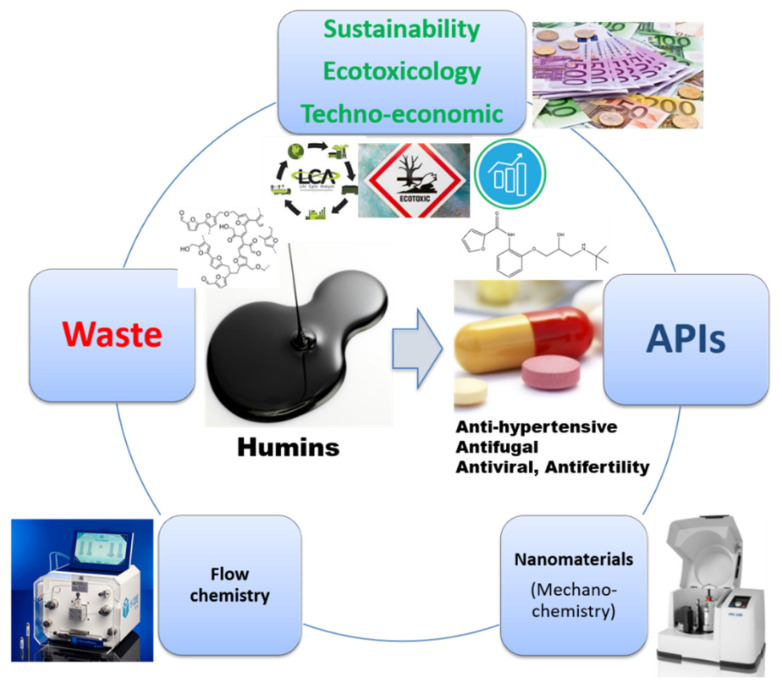
Project concept: from waste to the next generation of bioactive furanics under sustainable premises. The synthesis of APIs from biomass-derived waste has the potential to be conducted using green technologies, including mechanochemistry, photocatalysis and continuous flow processes (flow chemistry).

**Scheme 3:**
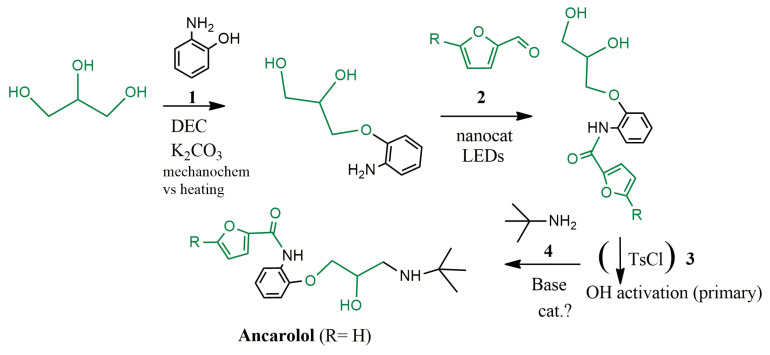
Proposed Ancarolol analogue syntheses from renewable feedstocks: humins and glycerol. DEC: Diethyl carbonate; K_2_CO_3_: potassium carbonate. Step 3 involves the addition of Tosyl chloride (TsCl) as an activating group for primary OH activation from the glycerol moiety.

**Scheme 4:**
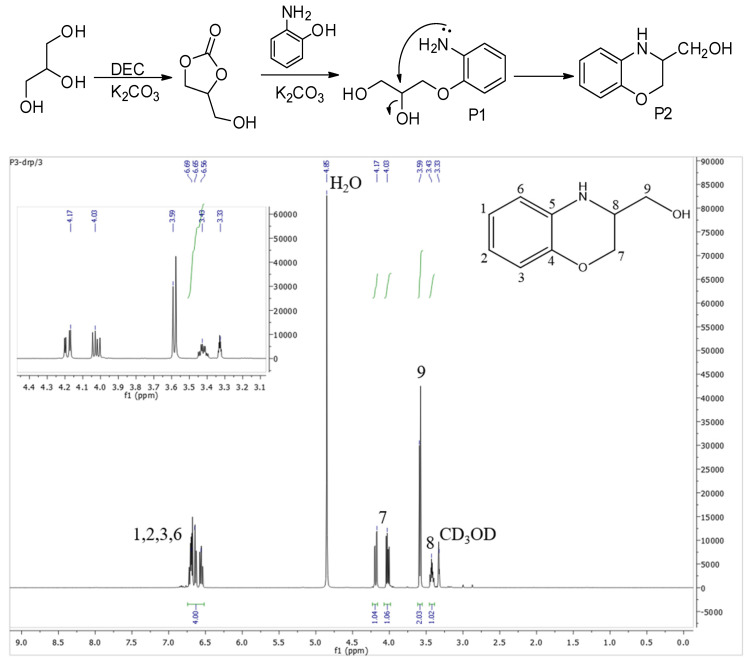
Plausible reaction mechanism for 3,4-dihydro-2*H*-benzo[*b*][1,4]-oxazin-3-yl)methanol formation from glycerol and 2-aminophenol (**top**). ^1^H-NMR spectrum of purified benzoxazine product (**bottom**). DEC: Diethyl carbonate; K_2_CO_3_: potassium carbonate.

**Scheme 5:**
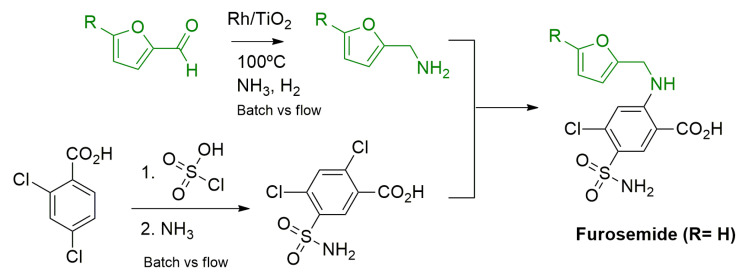
Proposed synthesis of Furosemide from furanics. Reactions performed in batch could be translated into more scalable continuous flow approaches.

## Data Availability

Not applicable.
